# 1874. Prior Screening for Latent Tuberculosis among Patients Diagnosed with Active Tuberculosis: Missed Opportunities?

**DOI:** 10.1093/ofid/ofad500.1702

**Published:** 2023-11-27

**Authors:** Saadiq Garba, Heidi Fischer, Lei Qian, Zhuoxin Li, Katia J Bruxvoort, Jacek Skarbinski, Jennifer H Ku, Bruno Lewin, Sally Shaw, Brigitte Spence, Sara Y Tartof

**Affiliations:** Kaiser Permanente Bernard J. Tyson School of Medicine, Pasadena, California; Kaiser Permanente Southern California, Pasadena, California; Kaiser Permanente Southern California, Pasadena, California; Kaiser Permanente Southern California, Pasadena, California; University of Alabama at Birmingham, Birmingham, Alabama; Kaiser Permanente Northern California, Oakland, California; Kaiser Permanente Southern California, Pasadena, California; Kaiser Permanente Department of Research and Evaluation, Pasadena, CA; Kaiser Permanente Southern California, Pasadena, California; Kaiser Permanente Southern California, Pasadena, California; Kaiser Permanente Southern California, Pasadena, California

## Abstract

**Background:**

California has the largest number of active tuberculosis (TB) cases in the US; more than 2 million Californians are estimated to have latent tuberculosis infection (LTBI), and 87% of TB cases in 2021 were attributed to LTBI reactivation. This study in a large California health system assessed missed opportunities for LTBI screening and treatment among patients with active TB.

**Methods:**

Kaiser Permanente Southern California patients who were ≥18 years with membership for ≥24 months from January 1, 2008 to December 31, 2019 were included. Receipt and result of prior LTBI test (tuberculosis skin test or interferon gamma release assay) or LTBI diagnosis code prior to active TB diagnosis was estimated among patients with observed active TB (confirmed by polymerase chain reaction and/or culture). LTBI testing/diagnosis < 60 days before observed active TB diagnosis was considered part of the active TB diagnostic process, and not part of LTBI screening. A range of hypothetical active TB cases prevented through current LTBI screening practices was estimated by multiplying literature supported LTBI reactivation rates by the number of treated LTBI positive patients. These hypothetical cases were categorized to have their first LTBI positive test/diagnosis ≥60 days prior to active TB.

**Results:**

A total of 1,289 patients with observed active TB were identified during the study period. Among them, 148 patients had a positive LTBI test/diagnosis and 84 patients had a negative LTBI test ≥60 days before active TB onset. There were 1,057 patients who either never had an LTBI test/diagnosis, or had an LTBI test/diagnosis <60 days before active TB onset who made up 82.0% of observed active TB cases (1,057/1,289). Adding the maximum estimate for possible prevented cases decreased the percentage of patients with TB who had never been screened for LTBI to 44.9% (1,057/1,289+1,066).

**Conclusion:**

Less than a fifth of patients were screened for LTBI prior to their active TB diagnosis during the study period. Even assuming the highest number of cases prevented through current screening, almost 45% of active TB patients were never screened for LTBI. Future work to elucidate gaps in LTBI screening practices and to identify opportunities to improve LTBI screening guidelines are needed.

**Disclosures:**

**Heidi Fischer, PhD**, Pfizer: Grant/Research Support **Lei Qian, PhD**, Dynavax: Grant/Research Support|GlaxoSmithKline: Grant/Research Support|Moderna: Grant/Research Support **Katia J. Bruxvoort, PhD, MPH**, Dynavax: Grant/Research Support|GlaxoSmithKline: Grant/Research Support|Moderna: Grant/Research Support|Pfizer: Grant/Research Support **Jacek Skarbinski, MD**, Genentech: Grant/Research Support|Gilead Sciences: Grant/Research Support **Jennifer H. Ku, PhD MPH**, GlaxoSmithKline: Grant/Research Support|Moderna: Grant/Research Support **Sara Y. Tartof, PhD MPH**, Genentech: Grant/Research Support|GSK: Grant/Research Support|Pfizer: Grant/Research Support|SPERO: Grant/Research Support

